# Examination of endobronchial ultrasound-guided transbronchial needle aspiration using a puncture needle with a side trap

**DOI:** 10.1038/s41598-021-89244-x

**Published:** 2021-05-07

**Authors:** Kazuhito Miyazaki, Yuya Hirasawa, Masaharu Aga, Naoto Aiko, Yusuke Hamakawa, Yuri Taniguchi, Yuki Misumi, Yoko Agemi, Tsuneo Shimokawa, Hiroyuki Hayashi, Katsuhiko Naoki, Hiroaki Okamoto

**Affiliations:** 1 Department of Respiratory Medicine, Yokohama Municipal Citizen’s Hospital, Yokohama, Kanagawa Japan; 2 Department of Pathology, Yokohama Municipal Citizen’s Hospital, Yokohama, Kanagawa Japan; 3 Department of Respiratory Medicine, Kitasato University School of Medicine, Sagamihara, Kanagawa Japan

**Keywords:** Lung cancer, Cancer screening, Predictive markers

## Abstract

Endobronchial ultrasound-guided transbronchial needle aspiration (EBUS-TBNA) is useful for diagnosing hilar and mediastinal lymph node enlargement; however, specimens obtained are often small and inadequate for pathologic diagnosis. In June 2017, EchoTip ProCore, a puncture needle with a side trap, was launched in Japan. In this single-center prospective interventional study, 57 patients with lymph nodes, intrapulmonary tumor or pleural mass were diagnosed using EBUS-TBNA with EchoTip ProCore between June 2017 and February 2020. EBUS-TBNA was performed for 57 patients and 53 patients had sufficient specimen for histologic diagnosis. The following pathologic subtypes were diagnosed: non-small cell lung cancer, 22; small cell lung cancer, 8; cancer of unknown primary, 2; neuroendocrine tumor (G2) recurrence, 1; lymphoma, 2; metastatic renal cell carcinoma, 3; thymoma recurrence, 1; sarcoidosis, 4; tuberculosis, 1; and non-malignancy, 9. In addition, the cytology showed Class V in 31 out of 57 cases (54.4%). In total, a definitive pathological diagnosis was obtained in 50 out of 57 cases (87.7%). The only complication was pneumonia caused by BAL simultaneously combined with EBUS-TBNA in one patient. Among 13 patients with inadequate specimens or without malignancy, only one patient was subsequently diagnosed with malignancy, and the median follow-up period was 300 days. EBUS-TBNA using EchoTip ProCore can obtain a sufficient specimen size for pathologic diagnosis.

## Introduction

Endobronchial ultrasound-guided transbronchial needle aspiration (EBUS-TBNA) is a useful diagnostic modality for thoracic lymphadenopathy, including primary lung cancer, malignant lymphoma, tuberculosis, and sarcoidosis^1–3^. Tissue and cytological specimens can be obtained using EBUS-TBNA. Notably, tissue samples obtained using this technique can undergo tests for epidermal growth factor receptor (EGFR) mutations and immunohistochemical screening for anaplastic lymphoma kinase (ALK) rearrangement in patients with non-small cell lung cancer (NSCLC). However, the specimens obtained are often small and inadequate for pathologic diagnosis. Specifically, only 66% (19/29) of specimens obtained using EBUS-TBNA at our hospital between 2015 and 2016 were adequate for histological diagnosis.

**Figure 1:**
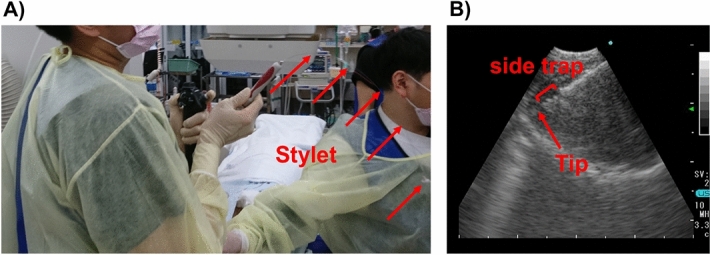
The slow-pull technique using EchoTip ProCore. (**A**) is where the slow pull method is performed in our hospital. B is an ultrasound image of EchoTip ProCore.

In June 2017, EchoTip ProCore Endobronchial HD Ultrasound Needle (EchoTip ProCore; Cook Medical, Bloomington, Indiana, United States), a puncture needle with a side trap that had been previously released for gastrointestinal endoscopes, was launched in Japan. EchoTip ProCore needles are available in different sizes, including 22- and 25-gauge needles. They are designed with a core trap proximal to the needle tip that receives the sample during fine-needle aspiration (FNA) through the needle tip (Fig. 1). Therefore, EchoTip ProCore is considered to obtain core biopsy for histologic evaluation rather than only cytological material, as in other needles^4^. This study aimed to investigate the diagnostic utility of EBUS-TBNA with EchoTip ProCore for mediastinal lymphadenopathy.

## Materials and methods

### Patients selection

This single-center prospective interventional study was conducted at the Department of Respiratory Medicine, Yokohama Municipal Citizen’s Hospital (Kanagawa, Japan). We included patients with radiological features of mediastinal or hilar lymphadenopathy who underwent EBUS-TBNA between June 2017 and February 2020. Chest computed tomography (CT) was performed before EBUS-TBNA, which revealed at least one enlarged mediastinal or hilar lymphadenopathy of > 10 mm in all patients except for two patients with a tumor around the trachea. Finally, we included 57 patients in this study.

### EBUS-TBNA

All enrolled patients underwent EBUS-TBNA using a convex endobronchial ultrasound bronchoscope probe (BF-UC260FW; Olympus, Tokyo, Japan) under fentanyl-induced sedation (0.025-0.05 mg/body) and midazolam (1–3 mg/body). A 22- and 25-gauge EchoTip ProCore Endobronchial HD Ultrasound Needle was used. The 25-gauge needle was employed if contrast-enhanced CT revealed blood vessel enrichment in a lymph node or if there was a high suspicion of hypervascular tumor metastasis, including renal cell carcinoma. We performed 2–4 punctures using the traditional vacuum syringe method and slow-pull technique. The specimens obtained with each needle pass were extracted to the dish by pushing the stylet and flushing the syringe with saline solution and air. Since rapid on-site cytological examination (ROSE) was not available at our hospital, the puncture was repeated up to 4 times when the volume of the collected sample was considered insufficient macroscopically. With regard to sample processing, each tissue from one puncture was placed in an embedding cassette and fixed in 10% neutral buffered formalin solution. A paraffin block of the sample was then prepared and histologically assessed. The remaining liquid components and small tissue fragments were collected with a dropper and placed in a spitz tube, a spitz tube was centrifuged at 2,000 rpm for 5 min and the sediment was subjected to liquid-based cytology (LBC) using CytoRich Red Preservative (Becton Dickinson, Franklin Lakes, New Jersey, United States) and assessed by cytologic diagnosis. At our hospital, clinical laboratory technicians (some of whom are cytotechnologists) process samples in the bronchoscope room, and the tissue was fixed with formalin immediately after all punctures were completed. Cytological samples were taken back to the laboratory with spitz tubes and sample processing was started immediately.

### Slow-pull technique

Originally, the slow-pull technique involved slow stylet retraction with needle fanning in the mass. Ten to twenty to-and-fro movements were made with minimal negative pressure provided by slow pulling of the stylet. The length of stylet retraction was about 1 m and the time of stylet retraction was from 40 to 60 s^5–7^. However, to obtain larger specimens, we used a modified version of the slow-pull technique. In short, although the stylet was slowly pulled out in 1 m, 10–30 to-and-fro movements with keeping negative pressure were repeated in every 5–10 cm up to the end.

### IRB approval

The study protocol was approved by the independent ethics committee of the institutional review board of Yokohama Municipal Citizen’s Hospital (No. 20-11-11). Informed consent was obtained in the form of opt-out on the web-site. Those who rejected were excluded. Topical subheadings are allowed. Authors must ensure that their Methods section includes adequate experimental and characterization data necessary for others in the field to reproduce their work.

### Statement of human rights

All procedures performed in studies involving human participants were in accordance with the ethical standards of the institutional and/or national research committee and with the 1964 Helsinki declaration and its later amendments or comparable ethical standards.

## Results

### Patients characteristics

Table 1 presents the baseline characteristics. This study enrolled 57 patients who underwent EBUS-TBNA using EchoTip ProCore between June 2017 and February 2020. We used 22- and 25-gauge EchoTip ProCore for 51 and 6 patients, respectively. The median age was 71 (range: 18–89) years, and 44 (77%) patients were male. The stations of the punctured lymph nodes were as follows: 1 for #2R; 20 for #4R; 3 for #4L; 26 for #7; 4 for #11 s; 2 for #11i; and 2 for #11L. In all patients, the lymph nodes were in contact with the trachea or bronchial wall. However, none of the tumor was visible in the central airway lumen when bronchoscope was performed. Additionally, a right upper lobe tumor and pleural mass were punctured in two separate patients (Table 2). Among 57 patients, 60 lymph nodes were punctured, with two lymph nodes being punctured in three patients. The median maximum diameter of the lymph nodes was 27 (range: 14–70) mm. Maximum diameter of right upper lobe mass was 21 mm, and pleural mass was 25 mm, respectively.

**Table 1:** Patients characteristics.

N = 57	N	(%)
Age, yr (Range)	71 (18–89)	
**Sex**		
Male	44	77.2
Female	13	22.8
**Punctured lymph nodes**		
#2R	1	1.7
#4R	20	33.3
#4L	3	5.3
#7	26	43.3
#11s	4	6.7
#11i	2	3.3
#11L	2	3.3
Right upper lobe mass	1	1.7
Pleural mass	1	1.7
**Median maximum diameter of lymph nodes**		
mm (Range)	27 (14–70)	
Maximum diameter of right upper lobe mass, mm	21	
Maximum diameter of pleural mass, mm	25	
**EchoTip ProCore**		
22G	51	89.5
25G	6	10.5

Baseline demographic and clinical characteristics of 57 patients who underwent EBUS-TBNA with EchoTip ProCore between June 2017 and February 2020.

**Table 2:** Pathological diagnosis.

	N	(%)
NSCLC	22	38.6
SCLC	8	14.0
RCC (metastasis)	3	5.3
CUP (Sq)	2	3.5
NET(G2) recurrence	1	1.8
lymphoma	2	3.5
thymoma recurrence	1	1.8
sarcoidosis	4	7.0
necrotic tissue	1	1.8
tuberculosis	1	1.8
non-malignancy	9	15.8
cyst	1	1.8
inadequate	2	3.5
Class V	31	54.4
Class IV	0	0
Class III	8	14.0
Class II	4	7.0
Class I	14	24.6

Pathological findings of tissue or cytological specimen obtained using EBUS-TBNA.

*NSCLC* non-small cell lung cancer, *SCLC* small cell lung cancer, *RCC* renal cell cancer, *CUP* cancer of unknown primary, *NET* neuroendocrine tumor, *Sq* squamous cell carcinoma, *Class I* Absence of atypical or abnormal cells, *Class II* Atypical cytology but no evidence of malignancy, *Class III* Cytology suggestive of, but not conclusive for malignancy, *Class IV* Cytology strongly suggestive of malignancy, *Class V* Cytology conclusive for malignancy.

### EBUS-TBNA

**Figure 2:**
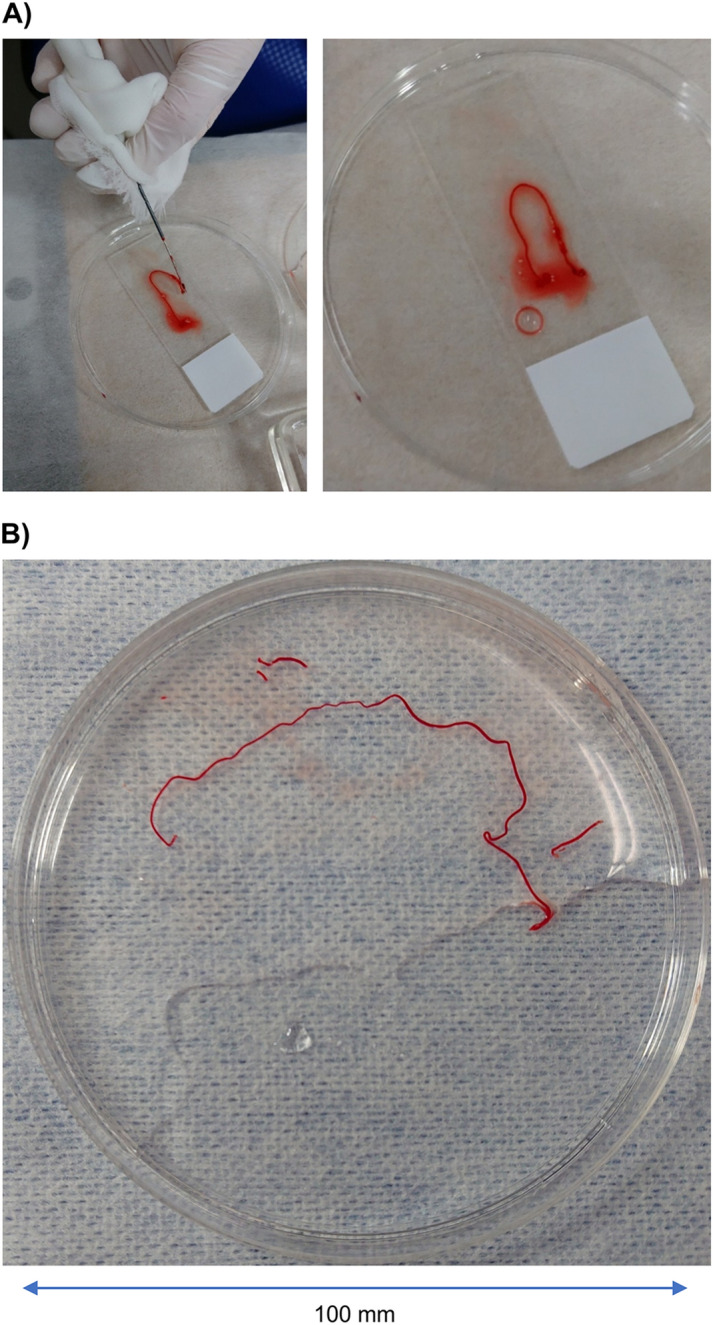
Tissue samples obtained using EchoTip ProCore. (**A**) presents a tissue sample obtained using a 22-gauge EchoTip ProCore where the #11i lymph node was punctured. (**B**) presents a tissue sample obtained using a 22-gauge EchoTip ProCore where the #4R lymph node was punctured in other cases. As shown here, this needle can obtain very large tissue samples.

**Figure 3:**
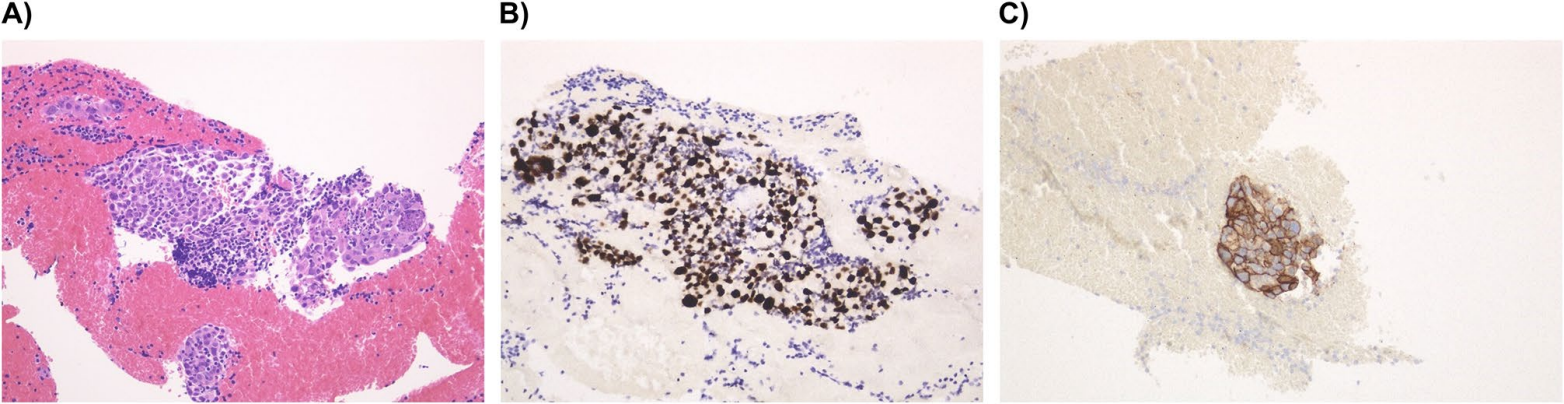
Pathological examination of one case of adenocarcinoma. (**A**) Hematoxylin–eosin staining. (**B**) Thyroid transcription factor-1 (TTF-1) staining using TTF-1 antibody. C) PD-L1 staining using PD-L1 IHC 22C3 PharmDx assay.

Figure 1 illustrates the slow-pull technique using EchoTip ProCore. As shown in Fig. 1(A), the puncture is repeated with gradual pulling out of the stylet. Figure 1B presents an ultrasound image of EchoTip ProCore. There is a side trap on the central tip side that shaves off the tissue. Figure 2 shows the tissue samples obtained using EchoTip ProCore. Figure 2A presents a tissue sample obtained using a 22-gauge EchoTip ProCore where the #11i lymph node was punctured. Hematoxylin–eosin staining showing large, strongly atypical cells, as well as positive TTF-1 staining, led to a histological diagnosis of metastatic lung adenocarcinoma. Subsequently, PD-L1 immunohistochemistry (IHC) revealed that 95% of the cells were positive; however, the cell number had been reduced by re-thinning (Fig. 3). Figure 2B presents details of the other patients. There was lymph node enlargement of the right supraclavicular fossa and mediastinum; subsequently, EBUS-TNBA with EchoTip ProCore was performed to differentiate among lymphoma, lymphoproliferative disease, and mediastinal lung cancer. A 22-gauge EchoTip ProCore was used for #4R lymph node puncture. The lymph node was soft, and the tissue was obtained using the slow-pull technique, which yielded a large tissue with a size > 10 cm, as shown in Fig. 2B. Pathological examination revealed an epithelioid granuloma; moreover, the interferon-gamma release assay for tuberculosis was positive; thus, the patient was diagnosed as having active tuberculosis.

As for the complications of EBUS-TBNA using EchoTip ProCore, none of the patients had massive bleeding, mediastinitis, or pneumothorax. Only one case of suspected sarcoidosis with bronchoalveolar lavage (BAL) developed pneumonia after bronchoscopy, probably owing to BAL, which was positive for the pneumococcal urinary antigen and was relieved by oral levofloxacin treatment.

### Pathological diagnosis

Among 57 patients, one had a cyst, one had necrotic tissue, and two had inadequate specimens; however, the remaining 53 (93%) patients had sufficient specimen for histologic diagnosis using EBUS-TBNA (Fig. 3). The following pathologic subtypes were diagnosed: NSCLC, 22; small cell lung cancer (SCLC), 8; cancer of unknown primary (squamous cell carcinoma), 2; neuroendocrine tumor (G2) with thymus origin recurrence, 1; lymphoma, 2; metastatic renal cell carcinoma, 3; thymoma recurrence, 1; sarcoidosis, 4; tuberculosis, 1; and non-malignancy, 9. Moreover, cytology revealed that 68% (39/57) of patients were class III or higher. In total, a definitive pathological diagnosis was obtained in 50 out of 57 cases (87.7%). A patient with necrosis on histological examination was diagnosed as having SCLC based on cytological examination. Two patients lacked a definitive diagnosis based on histological examination; however, cytological examination was indicative of class III, and a lymphoma was detected on surgical lymph node biopsy. One of the 10 patients with class II according to cytological examination was diagnosed as Hodgkin's lymphoma based on surgical lymph node biopsy. Among the remaining patients, 8 were followed up, with 1 being excluded after being requested to be examined in another hospital. The median observation period for the 8 patients was 300 (range 223–665) days. However, for one patient who underwent lung cancer surgery, CT and positron emission tomography did not reveal changes in the punctured lymph nodes; however, brain recurrence occurred.

Histological diagnosis revealed lung cancer in 97% (30/31) of patients, and EBUS-TBNA with EchoTip ProCore was used for diagnosis in all patients, including cytological diagnosis.

### PD-L1 immunohistochemistry

The PD-L1 status was measured based on IHC using the PD-L1 IHC 22C3 PharmDx assay (Agilent Technologies, Santa Clara, California, United states). Among 22 NSCLC patients, IHC of PD-L1 using the 22C3 antibody was possible in 19 patients except for 1 patient each where EBUS-TBNA was performed to differentiate between lung and breast cancer recurrence for staging purposes and to search for T790M mutation in EGFR-positive NSCLC, respectively. The PD-L1 tumor protein scores (TPSs) were ≥ 50%, 1–49%, and < 1% in 8 (42%), 5 (26%), and 6 (32%) patients, respectively (Table 3).

**Table 3:** PD-L1 status of NSCLC.

PD-L1 (TPS)	N	(%)
50% >	8	42.0
1–49%	5	26.0
1% <	6	32.0

PD-L1 status non-small cell lung cancer was examined through IHC using the PD-L1 IHC 22C3 PharmDx assay.

*TPS* tumor proportion score.

### Driver mutation test

At the time of study initiation, EGFR, ALK and PD-L1 examinations were approved in Japan. Thereafter ROS1 and comprehensive genome sequencing (Oncomine test) were sequentially approved between the study period. Therefore, the frequency of each biomarker test was different among the patients.

Among 22 NSCLC patients, 17 cases were adenocarcinoma, 4 cases were squamous cell carcinoma (Sq), 1 case was non-small cell lung carcinoma, not otherwise specified (NSCLC, NOS). In 18 cases of non-Sq-NSCLC, gene testing was performed except for one case of staging. EGFR mutations were wild/mutation/not examined: 12/4/1, ALK rearrangement were wild/ rearrangement/not examined: 15/0/2, and ROS1 rearrangement were wild/rearrangement/not determined but examined/not examined: 9/0/5/3 cases (Table 4). Of the above results, Oncomine Dx. Target Test (Thermo Fisher Scientific, San Jose, CA, United States) was used in only one case.

**Table 4:** Driver mutation status of non-Sq-NSCLC.

N = 17	N	(%)
**EGFR**		
Wild type	12	70.6
Mutation	4	23.5
Not examined	1	5.9
**ALK**		
Wild type	15	88.2
Rearrangement	0	0
Not examined	2	11.8
**ROS1**		
Wild type	9	52.9
Rearrangement	0	0
Not determined but examined	5	29.4
Not examined	3	17.6

EGFR mutation, ALK rearrangement, ROS1 rearrangement status of non squamous non-small cell lung cancer was examined.

## Discussion

Endoscopic ultrasound-FNA using EchoTip ProCore was primarily employed in gastroenterology (especially in pancreatic cancer) and rarely in the respiratory field^8,9^. To our knowledge, this is the first report regarding the large-scale utility of EBUS-TBNA with EchoTip ProCore. Bronchoscopic examination is widely used for initial diagnosis. Specifically, EBUS-TBNA is considered the first choice for mediastinal lymphadenopathy given that it has an almost similar diagnosis rate as mediastinoscopy and is minimally invasive^10^.

Treatment of advanced lung cancer has dramatically changed since the discovery of the EGFR gene mutation in the 2000s and various driver gene mutations, including ALK rearrangement^11–13^. Furthermore, nivolumab, which is a programmed death-1 antibody, was approved in December 2015 in Japan; subsequently, multiple immune checkpoint inhibitors have been approved^14–17^. The PD-L1 expression rate in tissues is an important predictor of the effect of immune checkpoint inhibitors^14,15^. Therefore, EBUS-TBNA with mediastinal lymphadenopathy has been a useful procedure for lung cancer. There was an increasing need for a reliable method to obtain tissue samples.

From 2015 to 2016, EBUS-TBNA was performed in 29 cases at our hospital; however, tissue diagnosis was possible in 19 cases (66%), and the diagnosis was confirmed using tissues in 10 cases (34.5%) and including cytology 13 cases (44.8%). In contrast, in the present study, tissue samples were available for 54 (94.7%) of 57 EBUS-TBNA cases using EchoTip ProCore, and a definitive diagnosis was obtained for 50 (87.7%) cases. A definite diagnosis was made for 30/31 (97%) lung cancer cases; a diagnosis was obtained for each of the 31 cases including cytological diagnosis. In the literatures, the diagnostic rate of EBUS-TBNA using a conventional puncture needle was reported to be about 51% for both 21-gauge and 22-gauge for mediastinal and hilar lymph nodes^18^. Other reports showed the diagnostic rate using conventional EBUS-TBNA methods were in the range of 61 to 80%^19,20^. Although the patient characteristics between our study and other reports were different, the diagnostic rate of current study seems to be superior than that of conventional EBUS-TBNA methods.

Although there was a difference between the number of cases that underwent EBUS-TBNA before and after the introduction of EchoTip ProCore (13.5 and 20.7 cases/year, respectively), there was no significant increase or decrease in the number of bronchoscopies in our hospital, suggesting an improvement in the tissue diagnosis rate after the introduction of EchoTip ProCore. Therefore, the introduction of EchoTip ProCore contributed to more accurate diagnoses. Three of 31 lung cancer cases were difficult to diagnose because of peripheral lesions that could not be pathologically diagnosed, and EBUS-TBNA was successfully used for tissue diagnosis. From this evidence, the diagnostic rate of EchoTip ProCore is superior to that of EBUS-TBNA using a standard needle. Regarding safety, only one of 57 patients who had punctured a cyst received prophylactic antimicrobials after EBUS-TBNA. None of the patients developed mediastinitis, and only one case of suspected sarcoidosis with BAL developed pneumonia after bronchoscopy, probably owing to BAL, which was positive for the pneumococcal urinary antigen and was relieved by oral levofloxacin treatment. No other complications were observed. The incidence of complications after EBUS-TBNA has been reported to be 0.15 to 0.46%^21,22^, and EchoTip ProCore was safe as it did not increase the incidence of complications. Furthermore, several studies have reported that samples obtained using EBUS-TBNA as a cell block can allow PD-L1 staining^23,24^. The present study shows that EBUS-TBNA with EchoTip ProCore can consistently yield large tissues suitable for direct immunostaining of PD-L1 (Fig. 3). Of course, tests for EGFR, ALK, and ROS1 were also available. Additionally, in one patient, Oncomine Dx. Target Test, which is the first FDA-approved next-generation sequencing-based companion diagnostic method for diagnosing EGFR mutation, ALK rearrangement, ROS1 rearrangement, and BRAF V600E mutation in patients with NSCLC, was performed without complications using our methods. Specifically, EBUS-TBNA with EchoTip ProCore can collect appropriate samples for next-generation sequencing examinations. Among 22 patients with NSCLC who could yield histological specimens, the PD-L1 TPS was successfully examined in 19 patients except for one patient for staging reasons, one for differentiation between breast and lung cancer recurrence, and one for T790M search purposes. This high success rate could contribute to the selection of more appropriate individual therapy for patients with NSCLC.

This study had some limitations. First, it was difficult to puncture small lymph nodes with EchoTip ProCore given its side core. Second, this was a small uncontrolled study, a single-center study and did not perform comparisons with other needles. However, as shown in Fig. 2, this needle allowed us to consistently obtain large tissue samples. Finally, it was unclear whether the superior diagnostic rate was attributed to the slow-pull technique or EchoTip ProCore. Although examples are not shown, we attempted EBUS-TBNA in a small number of patients at our institution with the slow-pull technique using the 22-gauge Vizishot2 needle (Olympus, Tokyo, Japan). However, tissue specimen collection was not as stable as with EchoTip ProCore. This indicates that the slow-pull technique using EchoTip ProCore may be more important.

In conclusion, our method allowed pathological diagnosis of 54 of 57 patients using histology, cytology, or both. The PD-L1 TPS was successfully examined in all 19 patients with NSCLC who could yield histological specimens and required examination. Additionally, none of the patients had complications. Among 13 patients with inadequate specimens or without malignancy, only one patient was subsequently diagnosed as having malignancy with a median follow-up period of 300 days. Therefore, EBUS-TBNA using a puncture needle with a side trap should be considered for patients with hilar and mediastinal lymphadenopathy in routine clinical practice.
